# Rare Case of Acute Pulmonary Thromboembolism After Internal Fixation of Multiple Rib Fractures

**DOI:** 10.1002/ccr3.70899

**Published:** 2025-09-21

**Authors:** Bao‐ping Xu, Hua‐min Wang, Ba‐yi Liu, Xiao‐tao Wang, Sen Zhu, Zhen‐jun Li, Dian‐feng Deng

**Affiliations:** ^1^ Department of Critical Care Medicine Zhongshan Hospital of Traditional Chinese Medicine Affiliated to Guangzhou University of Traditional Chinese Medicine Zhongshan China; ^2^ Department of TCM Ehu Branch of Xishan People's Hospital of Wuxi City Wuxi China; ^3^ Department of Orthopedics Gongli Hospital Affiliated to Naval Military Medical University Shanghai China; ^4^ Department of Orthopedics, Gansu Provincial Hospital of Traditional Chinese Medicine Affiliated to Gansu University of Traditional Chinese Medicine Lanzhou China; ^5^ Department of Internal Medicine Ehu Branch of Xishan People's Hospital of Wuxi City Wuxi China

**Keywords:** case report, multiple rib fractures, pulmonary thromboembolism, surgical stabilization of rib fractures

## Abstract

Acute pulmonary thromboembolism (PTE) after isolated multiple rib fractures is rare; although surgical stabilization of rib fractures (SSRF) is a relatively safe and effective treatment for multiple rib fractures with dislocation, perioperative PTE should be given sufficient attention by clinicians. The standardized prevention of perioperative venous thromboembolism for multiple rib fractures requires further research due to the lack of high‐quality clinical evidence support.

## 1 Introduction

Multiple rib fractures have a high mortality rate and are one of the most common incident clinical fractures in emergency trauma patients [1, 2]. Approximately 13% of patients with rib fractures experience one or more complications, including pneumonia, respiratory failure, pneumothorax, pleural effusion, empyema, necrotizing pneumonia, and lung abscesses [3]. Surgical stabilization of rib fractures (SSRF) has been considered a safe and effective method to treat multiple rib fractures, especially for patients with displaced rib fractures. In these cases, SSRF has been shown to have improved survival [2, 4]. To our knowledge, acute pulmonary thromboembolism (PTE) has not been reported in the literature after surgical fixation of isolated multiple rib fractures. We report a case of a 64‐year‐old male who developed acute PTE after internal fixation of the rib fractures, and his symptoms improved significantly after anticoagulant therapy with enoxaparin. To the best of our knowledge, such a case has been reported in the English literature, and our case may provide some limited evidence for clinicians. Future studies are required to standardize the management of such complications in patients with multiple rib fractures.

## 2 Case Presentation

### 2.1 Case History/Examination

A 64‐year‐old male was admitted to our hospital with persistent chest pain for 3 h after a traffic accident in March 2022. The patient had a 3‐year history of hypertension and is not currently taking any medications. The patient's temperature was 36.7°C, his heart rate was 92 beats per minute, his respiratory rate was 22 breaths per minute, his blood pressure was 111/72 mm of Hg, and his oxygen saturation was 94% on ambient air. He had multiple skin abrasions, soft tissue contusions, and chest wall deformity. The clinical examination of the chest revealed moist rales in both lungs, paradoxical breathing, and diffuse tenderness in the right ribs. Heart, abdomen, and nervous system examinations revealed no abnormalities. The peripheral circulation and sensory functions of the extremities were normal, but multiple skin contusions exist on the limbs.

**FIGURE 1:**
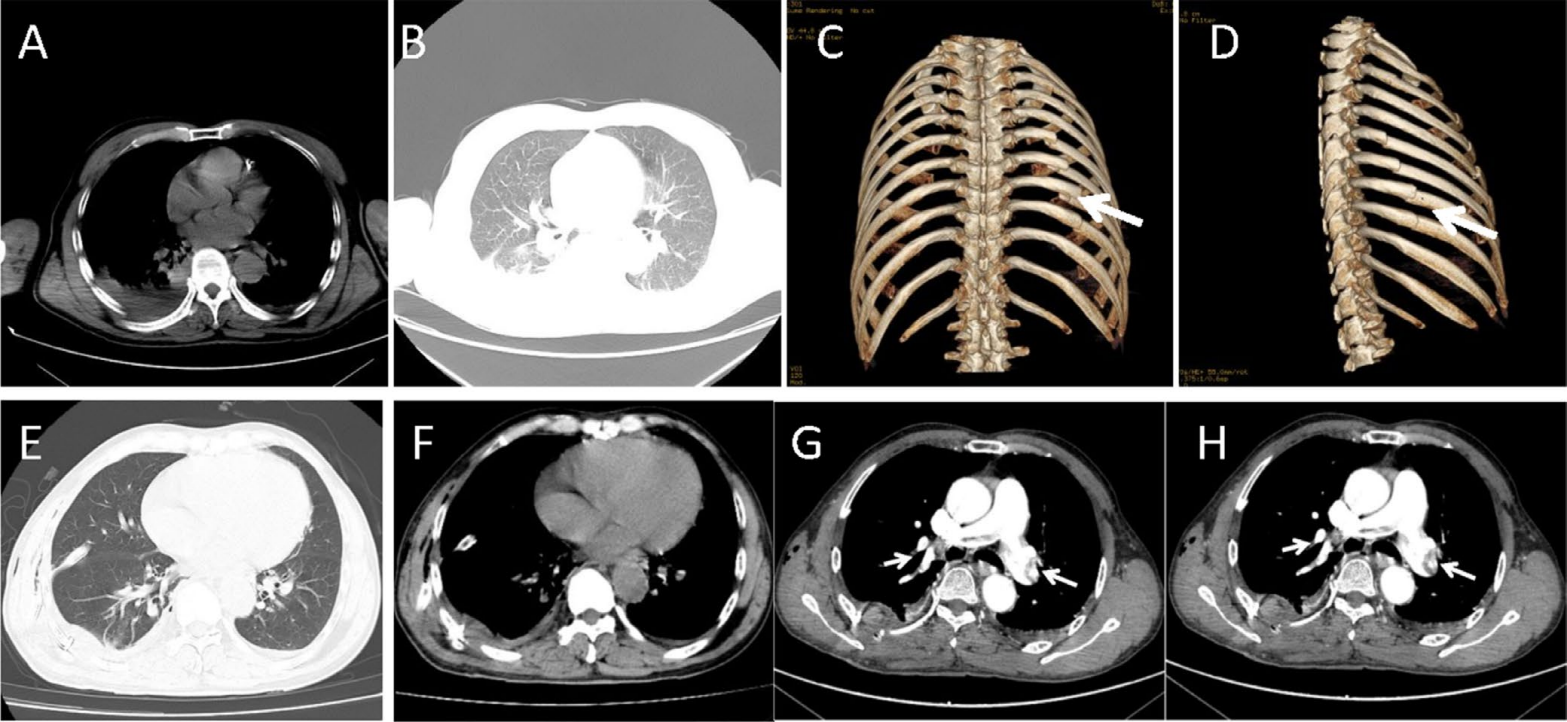
(A) A thoracic CT scan exhibited bilateral pleural effusion with apparent compression and incomplete expansion of the lower lobe of the right lung. (B) The patchy and strip‐like increased density on the back of both lungs was found in the chest CT. (C, D) 3D reconstruction of the chest CT revealed multiple right fractures of the 2nd–10th ribs on the right side with partial dislocation. (E, F) CT of the chest indicated a significant decrease in pleural effusion and a small amount of inflammation in both lungs. (G, H) Pulmonary artery angiography of the CT revealed multiple pulmonary embolisms at the distal end of the left and right main pulmonary arteries and branches of each lobe. Chest CT revealed exudative lesions of both lungs with atelectasis in the lower lobe of the left lung and a small amount of pleural effusion in the lungs.

Complete blood count revealed a white blood cell count of 4.70 × 10^9^/L (normal range: 4–10 × 10^9^/L), neutrophil count of 3.29 × 10^9^/L (normal range: 2–7.5 × 10^9^/L), hemoglobin level of 135.00 g/L (normal range: 110–160 g/L), and blood platelet count of 156.00 × 10^9^/L (normal range: 100–300 × 10^9^/L). His highly sensitive C‐reactive protein (HsCRP) concentration was 31.73 mg/L (normal range: 0–5 mg/L), and his initial D‐dimer level was 1.87 μg/mL (normal range: 0–0.5 μg/mL). Liver and kidney function, blood lipids, electrolytes, and procalcitonin were normal. The electrocardiogram revealed sinus rhythm. Cranial and abdominal computed tomography (CT) scan revealed no abnormalities. CT chest revealed lower lung infection with pleural effusion bilaterally with apparent compression and incomplete expansion on the lower lobe of the right lung. Three‐dimensional reconstructed image of ribs revealed multiple fractures of the 2nd–10th ribs on the right side with partial dislocation (Figure 1A–D).

## 3 Methods

He was initially treated with budesonide suspension atomizing inhalation, expectorant, analgesic, and intravenous fluids for 1 week before the operation. On March 14, 2022, he underwent thoracoscopic exploration combined with internal fixation of the right fracture of the 6th–8th ribs and recovered after surgery. General analgesics, infection prevention, phlegm elimination, and other symptomatic treatments were performed postoperatively. After surgery on March 16, 2022, he began anticoagulant therapy to prevent deep vein thrombosis with low molecular weight heparin sodium 2500 units once daily. On the third day after surgery (17 March 2022), a chest CT scan (Figure 1E,F) re‐examined a significant decrease in pleural effusion and a small amount of inflammation in both lungs after treatment. However, the chest CT scan still revealed signs of atelectasis. On March 18, 2022, laboratory investigations revealed a serum HsCRP concentration of 34.67 mg/L and serum D‐Dimer concentration of 2.97 μg/mL. Routine blood tests, renal function, electrolytes, and other blood investigation results were within the normal range. The patient developed sudden dyspnea and chest pain while using the toilet in the afternoon of the same day. The clinical examination indicated that the patient had an oxygen saturation (SaO_2_) of 82% with nasal oxygen (4 L/min), his pulse rate was 121/min, his respiratory rate was 30/min, and his blood pressure was 131/68 mm of Hg. The myocardial enzyme profile and troponin analysis exhibited no abnormalities. Computer tomography pulmonary angiography (CTPA) revealed multiple filling defects in the bilateral main pulmonary arteries and their branches (Figure 1G,H). He was transferred to the intensive care unit, where rapid bedside color ultrasonography of the arteries and veins in both lower extremities revealed no thrombus or other abnormalities. The patient was classified as medium‐low risk of PTE and was given a subcutaneous injection of 60 mg enoxaparin every 12 h and nasal high‐flow oxygen (FiO_2_: 60%, flow rate: 60 L/min). The peripheral capillary oxygen saturation rose to 94%. Coagulation function analysis on March 19, 2022, demonstrated that prothrombin time (PT) was 11.20 s, activated partial thromboplastin time (APTT) was 23.60 s, D‐Dimer was 2.53 μg/mL, and other values were normal.

He recovered gradually, and the patient was transferred to the general ward with noticeable relief of symptoms on March 22, 2022, with no chest pain or significant dyspnea. His oxygen saturation was 96% with nasal oxygen (2 L/min). He remained on the anticoagulant treatment with enoxaparin for 10 days. Coagulation function analysis indicated that D‐Dimer was 0.41 μg/mL on March 27, 2022. Table 1 shows the laboratory results of the patient obtained during the hospital stay.

**TABLE 1:** Laboratory tests of the patient during hospitalization performed along with timeline.

Lab tests	March 08	March 18	March 19	March 24	March 27	Normal range
White blood cell count	4.70	5.90	6.10	5.30	4.90	4–10 × 10^9^/L
Neutrophil count	3.29	3.74	3.98	3.54	3.34	2–7.5 × 10^9^/L
Hemoglobin	135	121	118	120	116	110–160 g/L
Blood platelet count	156	143	165	162	135	100–300 × 10^9^/L
C‐reactive protein	31.73	34.60	45.54	21.51	15.61	0–5 mg/L
D‐dimer	1.87	2.97	2.53	1.29	0.41	0–0.5 μg/mL
Prothrombin time	13.20	12.10	11.20	14.50	13.70	8–14 s
Activated partial thromboplastin time	22.40	20.50	23.60	43.50	38.60	20–40 s

## 4 Conclusion and Results

**FIGURE 2:**
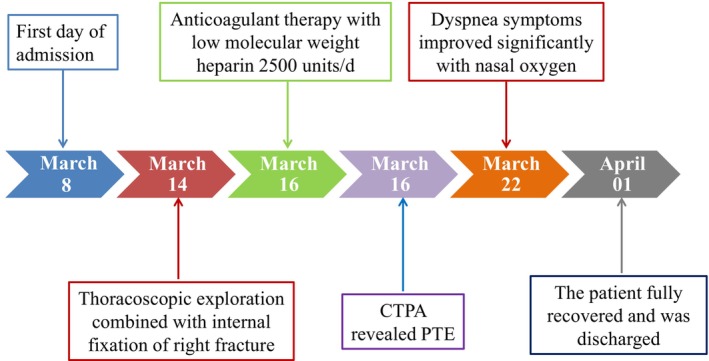
A timeline of management during patient hospitalization.

The patient was discharged from the hospital on April 01, 2022, and switched to oral anticoagulant therapy with rivaroxaban (20 mg/day). The patient was followed up for 3 months after discharge. CTPA revealed the thrombus had disappeared, and the D‐Dimer level was normal. A timeline of the case report is presented in Figure 2.

## 5 Discussion

A rib fracture is one of the most common injuries in blunt trauma, occurring in approximately 10% of all trauma patients [5]. Multiple rib fractures can cause chest instability and affect respiratory function; surgical treatment can stabilize ribs, restore lung capacity, and reduce postoperative complications and mortality of rib fractures [3, 6]. Research findings imply that surgical treatment can provide strong fixation support and faster recovery than conservative treatment, with a lower risk of complications and a better prognosis in patients with multiple rib fractures [7, 8]. Therefore, SSRF should be the preferred treatment modality for patients with multiple rib fractures, especially displaced ones [5]. This patient's 3D rib reconstruction revealed multiple rib fractures ranging from 2 to 10, with partial dislocation and pulmonary atelectasis. Surgical treatment is better than conservative treatment, so rib fixation surgery is performed after preoperative examinations. The patients recover without dyspnea, chest tightness, and chest pain within 3 days.

Complications, such as pneumothorax, pleural effusion, pulmonary contusion, pneumonia, atelectasis, respiratory failure, and vascular and solid‐organ injury, are common in multiple rib fractures and have a high mortality rate [3, 9]. The number of fractured ribs was associated with these complications, and as the number of rib fractures increased, so did mortality [9]. Additionally, there are several uncommon complications of rib fractures, such as delayed descending aortic tear [10], abdominal aortic injury [11], and gastrointestinal perforation [12]. PTE after surgery is rare for isolated multiple rib fractures, and there is limited literature. Therefore, clinicians may lack enough understanding of this uncommon complication and cannot conduct early standardized treatment to prevent acute PTE.

Venous thromboembolism (VTE), including deep vein thrombosis (DVT) and PTE, is one of the most common complications after orthopedic and general surgery, with high morbidity and mortality [13, 14]. VTE increases with age among adult trauma patients; despite significant advances in diagnosis and management, acute PTE remains one of the important causes of death [15, 16].

High‐level studies have been carefully conducted, and guidelines have been well established to prevent DVT and PTE in orthopedic and non‐orthopedic surgery patients [13]. The American Society of Thoracic Surgeons has established guidelines for preventing DVT in patients undergoing orthopedic surgery and standardized guidance on risk assessment, intervention method selection, and DVT treatment courses. However, the guidelines' primary target population is patients with hip fractures, total hip replacement, and knee replacement, and it excludes patients with rib fractures [17]. The American College of Chest Physicians also has guidelines for preventing DVT in non‐orthopedic surgery, including general surgery, neurosurgery, abdominal surgery, oncology, and thoracic surgery. The guidelines have been considered for preventing DVT after thoracic surgery and designated detailed guidelines. However, the guidelines for preventing perioperative DVT in thoracic surgery are primarily based on studies of patients undergoing tumor and cardiac surgery. There is a lack of research evidence and comprehensive and in‐depth knowledge regarding preventing perioperative DVT in rib fractures [18]. There are currently limited studies on perioperative VTE, including DVT and PTE for multiple rib fractures. No studies have been published describing chemoprophylaxis and preventing subsequent VTE for SSRF. Prevention of perioperative VTE for multiple rib fractures is based primarily on clinicians' limited experience. There are insufficient existing data and published literature to guide the standardized management of perioperative DVT for multiple rib fractures. Based on the existing expert consensus on the surgical treatment of rib fractures, it is recommended to routinely use low molecular weight heparin (LMWH) 30 mg twice a day to prevent DVT after SSRF [5], but there is no conclusive evidence regarding the need for anticoagulant therapy before the operation for rib fractures. The optimal time to initiate anticoagulant therapy after rib fracture surgery remains controversial. Although the expert consensus suggests using LMWH to reduce the risk of VTE within 24 h after SSRF is the appropriate time, the evidence is still limited [5, 19]. Furthermore, research findings suggest that LMWH administration should be delayed for 24 h following the placement of an epidural catheter; premature anticoagulation may cause rare complications, such as catastrophic intraspinal bleeding and spinal hematoma [20].

In this case report, the D‐Dimer level was high before surgery, indicating a hypercoagulable state of the blood. Considering the risk of bleeding, only physical measures were taken to prevent DVT, with no drug prophylaxis. The patient's D‐Dimer level remained high after SSRF, and several studies revealed that a high D‐Dimer level was a high‐risk predictor of DVT after thoracic surgery [21]. However, postoperative anticoagulant therapy was delayed until 48 h after surgery due to the excessive caution of surgeons. Although no complications, such as bleeding, occurred as expected, the patient developed an unexpected acute PTE 4 days after SSRF. Therefore, it is speculated that delayed anticoagulant timing may be an important cause of postoperative PTE.

The patient was treated with low molecular weight heparin sodium (2500 IU, qd) on the third postoperative day to prevent VTE. However, according to the current expert consensus in the surgical treatment of rib fractures, only half of the recommended daily dose of low molecular weight heparin sodium is insufficient. His serum D‐Dimer levels were 2.97 μg/mL on the fourth day after surgery, indicating that the blood was still hypercoagulable, implying that insufficient doses of anticoagulant drugs may be another important cause of PTE.

Risk stratification was classified by hemodynamic status and the simplified Pulmonary Embolism Severity Index score according to the 2014 European Society of Cardiology/European Respiratory Society guidelines [17]. In this case, the patient was evaluated as low‐risk, and anticoagulation was the most frequently adopted initial therapy [16]. After 2 weeks of subcutaneous enoxaparin, his symptoms were significantly relieved, with no chest tightness, pain, dyspnea, or other symptoms. After discharge, the anticoagulant therapy was changed to rivaroxaban for 3 months. Patients could not have their CTPA reviewed due to the COVID‐19 pandemic, so follow‐up imaging evaluation was lacking.

## Author Contributions

**Bao‐ping Xu:** conceptualization, writing – original draft. **Hua‐min Wang:** conceptualization, data curation, project administration, writing – review and editing. **Ba‐yi Liu:** conceptualization, data curation, project administration. **Xiao‐tao Wang:** writing – review and editing. **Sen Zhu:** writing – review and editing. **Zhen‐jun Li:** data curation, project administration. **Dian‐feng Deng:** conceptualization, writing – review and editing.

## Ethics Statement

Written informed consent for publication of their clinical details and/or clinical images was obtained from the patient.

## Consent

Written informed consent was obtained from the patient to publish this report in accordance with the journal's patient consent policy.

## Conflicts of Interest

The authors declare no conflicts of interest.

## Data Availability

The data were available upon appropriate requests from the corresponding author.
